# Using Inertial Measurement Units and Electromyography to Quantify Movement during Action Research Arm Test Execution

**DOI:** 10.3390/s18092767

**Published:** 2018-08-22

**Authors:** Eva Repnik, Urška Puh, Nika Goljar, Marko Munih, Matjaž Mihelj

**Affiliations:** 1 Faculty of Electrical Engineering, University of Ljubljana, Tržaška cesta 25, 1000 Ljubljana, Slovenia; marko.munih@robo.fe.uni-lj.si (M.M.); matjaz.mihelj@robo.fe.uni-lj.si (M.M.); 2 Faculty of Health Sciences, University of Ljubljana, Zdravstvena pot 5, 1000 Ljubljana, Slovenia; urska.puh@zf.uni-lj.si; 3 The University Rehabilitation Institute, Republic of Slovenia, Linhartova 51, 1000 Ljubljana, Slovenia; nika.goljar@ir-rs.si

**Keywords:** stroke, upper-limb movement, quantification, ARAT, inertial measurement unit, electromyography

## Abstract

In patients after stroke, ability of the upper limb is commonly assessed with standardised clinical tests that provide a complete upper limb assessment. This paper presents quantification of upper limb movement during the execution of Action research arm test (ARAT) using a wearable system of inertial measurement units (IMU) for kinematic quantification and electromyography (EMG) sensors for muscle activity analysis. The test was executed with each arm by a group of healthy subjects and a group of patients after stroke allocated into subgroups based on their clinical scores. Tasks were segmented into movement and manipulation phases. Each movement phase was quantified with a set of five parameters: movement time, movement smoothness, hand trajectory similarity, trunk stability, and muscle activity for grasping. Parameters vary between subject groups, between tasks, and between task phases. Statistically significant differences were observed between patient groups that obtained different clinical scores, between healthy subjects and patients, and between the unaffected and the affected arm unless the affected arm shows normal performance. Movement quantification enables differentiation between different subject groups within movement phases as well as for the complete task. Spearman’s rank correlation coefficient shows strong correlations between patient’s ARAT scores and movement time as well as movement smoothness. Weak to moderate correlations were observed for parameters that describe hand trajectory similarity and trunk stability. Muscle activity correlates well with grasping activity and the level of grasping force in all groups.

## 1. Introduction

Stroke is one of the leading causes of disability worldwide and the most common deficit after stroke is hemiparesis of the upper limb [1]. Muscle weakness and contracture, changes in muscle tone, joint laxity, impaired motor control, or other impairments caused by stroke induce disabilities in common activities such as reaching, picking up objects and holding onto objects [1]. Consequently, functional independence in performing activities of daily living and quality of life are reduced.

One of the most commonly used upper limb outcome measures at the activity level in stroke rehabilitation studies is the Action research arm test (ARAT) [2]. Due to the level of measurement, reliability and validity, and ability of minimal clinically important difference to capture gains associated with improved function, the panel of nine experts recommended its use for motor function intervention trials in population with chronic stroke [3]. The ability and quality of the tasks performance is graded on the ordinal scale, according to the set criteria by observation of a physiotherapist or occupational therapist. Each task is evaluated as a whole and the assessment does not provide evaluation of phases of the tasks (e.g., reaching, manipulation with object, and transfer).

In the last years, use of wearable systems with different sensors to measure upper limb kinematics gained significant interest [4,5,6,7]. Sensory systems are commonly used to observe and analyse human posture and movement [8]. Electromyography (EMG) is used for myoelectric control of upper limb prosthesis [9], and can be used for recognizing hand gestures [10] or as an assessment tool for spasticity after stroke [11]. Inertial measurement units (IMU) [12] and accelerometers [13] were used for objective arm performance measurement and statistical features from accelerometers data were used to predict motor function scores while repeatedly performing different tasks [14] and to assess quality of movement [15]. For objective assessment of spasticity in patients after stroke, IMU sensors and EMG electrodes were combined into an upper limb model, where IMUs were used for computing joint torques through inverse dynamics and EMG activities were used for comparison to the upper limb model predictions [16]. In some previous studies, sensors and other methods were already used to quantify standardized clinical tests. While executing ARAT, camera motion analysis system was used for movement time evaluation and end-point displacement [17], and from a single IMU on the wrist, duration of tasks and phases (reaching, manipulation, transfer, release, return) and jerk index were calculated [18]. IMU on the wrist was used to measure movement time while executing Wolf motor function test (WMFT) tasks [5,19] and jerk, power spectrum analysis, and approximate entropy of accelerometer data were calculated. Accelerometers were used to capture motion sample while executing WMFT and for automatic estimation of Fugl–Meyer assessment score using the kinematic features extracted from the sensor output, and thus proved the validity of using IMU as a sampling tool for clinical assessment [20]. During execution of WMFT tasks, IMU on the wrist, forearm and upper arm were used from which root mean square (RMS) value of accelerometer data, the RMS value of its derivative and the approximate entropy were calculated [21]. IMU and EMG sensors on the forearm were used while performing 11 tasks of Fugl–Meyer assessment scale to calculate their time duration and EMG and IMU power distribution, as well as accelerometer/gyroscope ratio to quantify the task pattern [22]. To the best of our knowledge, no study with multiple IMU and EMG sensors exists for assessment of upper limb and trunk movement during ARAT execution.

The aim of this study was to measure and quantify upper limb and trunk movement while executing ARAT motor tasks using wearable IMU and EMG sensory systems. The movement was quantified with five parameters that are associated with clinical assessment: movement time, movement smoothness, similarity of hand trajectories, trunk stability, and fingers and wrist muscle activity. While a therapist can reliably measure movement time, other movement characteristics are subjectively assessed from visual observations and finally combined with movement time into a single clinical score. Therefore, this study also investigated relations between the obtained numerical parameters and the corresponding clinical scores. Analysis was performed with healthy subjects and with patients after stroke with their affected and unaffected upper limbs.

## 2. Methodology

### 2.1. Participants

Patients who had been in the rehabilitation program after their strokes were recruited consecutively with the following criteria. They included those with the first middle cerebral artery stroke, with a partially impaired upper limb motor function (ability to grasp and lift an object from a table and ability to place the forearm on a table from starting position hands in lap), and no severe cognitive (mini mental state examination >25), somatosensory, visual or hearing impairment. Patients with previous musculoskeletal or nervous system impairment of the upper limbs or with poor general health condition (i.e., heart or respiratory problems, eating disorder, infective diseases and other health conditions which are not directly related to stroke but would prevent the patient to participate) were excluded.

This study included 28 participants after stroke (64% males) aged 38–74 years (mean 57; SD 9.1) and 5–80 weeks after stroke (mean 25; SD 17.3). Approximately half of the participants (53%) had left upper limb hemiparesis and the rest had right upper limb hemiparesis; all were right-handed before the stroke.

Healthy subjects were recruited according to convenience sampling from the laboratory staff. This study included 12 healthy participants (75% males) aged 26–55 years (mean 36; SD 8); all but one were right-handed.

All participants signed informed consent. This study was approved by the Republic of Slovenia national medical ethics committee and the University rehabilitation institute, Republic of Slovenia ethics committee (80/03/15).

### 2.2. Equipment and Procedure

**Figure 1:**
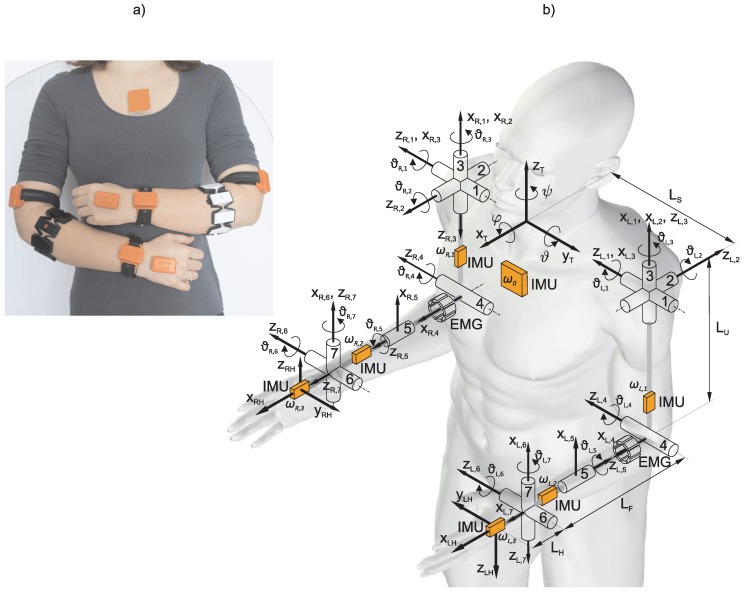
Wearable sensors and arm kinematics: (**a**) wearable system attached on a subject; and (**b**) kinematic model of the trunk and arms with joints (cylinders), segments and coordinate systems. Orange boxes represent IMU sensors, and the two segmented cylinders represent armband EMG electrodes.

Upper limb movement was measured with a wearable sensory system of seven wireless IMU sensors, consisting of a triad of magnetometers, a triad of accelerometers, a triad of gyroscopes and two EMG armbands (Myo armbands from Thalmic labs). The IMU sensors were attached, one on the dorsal side of each hand, wrist, upper arm and one on the sternum (the trunk sensor). Configuration of the wearable system is presented in Figure 1. The EMG armband is composed of eight stainless steel EMG electrodes and acquires EMG signals with sampling frequency of 200 Hz and transfers the data via Bluetooth to a computer. Standard material for ARAT, and templates on the table to specify the location of objects and start and end points for each task were used.

Equipped with wearable sensory system, the participants executed ARAT [23,24] tasks according to the standard protocols. Both upper limbs were tested in each participant. For all patients, concomitantly to kinematic and EMG measurements, the physiotherapist evaluated the tasks performance, giving clinical scores.

#### Action Research Arm Test (ARAT)

The ARAT [23,24] consists of 19 movement tasks divided into 4 subtests (grasp, grip, pinch, and gross arm movement). The first three subtests assess patient’s ability to pick-up objects of different sizes, shape, and weight (e.g., blocks, cricket ball, tube, marble, and bearing ball) from a table and carry them to a specific location (e.g., on the shelf), release the object and return the hand to the starting position. The last subtest assesses the movement of the entire upper limb. In each subtest, tasks are hierarchically ordered by difficulty. The ability to perform and quality of movement for each of the tasks are rated on a 4-point scale: Score 0 (not able to perform any part of the task); Score 1 (could only partially perform the task); Score 2 (could complete the task but took abnormally long (5 to 60 s) or had a great difficulty or the trunk loses contact with the back of the chair); and Score 3 (normal movement performance within 5 s).

### 2.3. Arm Kinematics

Configuration of the wearable system is presented in Figure 1 together with the schematic representation of relevant body segments and joints. Orange boxes represent IMU sensors and the two segmented cylinders represent armband EMG electrodes. Relevant for the analysis are the trunk, which represents the reference frame for analysis, and the two arms. The posture in Figure 1 defines zero values for all joint angles. In the presented kinematic model, the arm joints are defined in the following order from proximal to distal (+ and − signs indicate positive and negative joint angles): (1) shoulder flexion (−)/extension (+); (2) shoulder abduction (−)/adduction (+); (3) shoulder internal (−)/external (+) rotation; (4) elbow flexion (+)/extension (−); (5) wrist pronation (−)/supination (+); (6) wrist ulnar (−)/radial (+) deviation; and (7) wrist flexion (+)/extension (−).

Kinematic quantities considered for the analysis of arm motor functions are joint angles, hand position and their time derivatives. The Denavit–Hartenberg notation [25] was used for computation of the kinematic model with coordinate systems as defined in Figure 1. Joint variables are numbered from 1 to 7 for each arm. Matrix describing the relative transformation between the two consecutive coordinated systems as a function of the joint angle $\vartheta_{(L,R),j}$ (subscripts (*L*,*R*) represent left and right arm) can be written as $T_{(L,R),j}^{j-1}\left(\vartheta_{(L,R),j}\right)$ and the pose of the hand $T_{(L,R)H}$ relative to the trunk $\left(x_{T},y_{T},z_{T}\right)$ can be obtained as the product of matrices

(1) $$T_{(L,R)H}=\prod_{j=1}^{7}T_{(L,R),j}^{j-1}\left(\vartheta_{(L,R),j}\right).$$

The equation applies to both arms by considering the respective joint angles. In the following analysis, we do not specifically differentiate between quantities for the left and right arm, thus subscripts *L* and *R* are omitted.

Joint angles are computed from IMU data based on the following methodology [26]. The upper limb is represented as a series of joints connected by segments. A gyroscope within the ${\mathrm{IMU}}_{i}$ returns a vector of angular velocities $\omega_{i}={\left[\begin{array}{ccc} \omega_{x_{i}} & \omega_{y_{i}} & \omega_{z_{i}} \end{array}\right]}^{T}$. We define the Jacobian matrix J that relates joint rotational speeds $\dot{\vartheta }={\left[\begin{array}{c} {\dot{\vartheta }}_{1}\ldots {\dot{\vartheta }}_{7} \end{array}\right]}^{T}$ to a vector of all angular velocities of individual segments as

(2) $$\left[\begin{array}{c} \omega_{1}\\ \omega_{2}\\ \omega_{3} \end{array}\right]=\Omega =J\dot{\vartheta },$$

where $\omega_{1}$ to $\omega_{3}$ are angular velocities measured by the gyroscopes in the three IMU sensors on the left or right arm. The Jacobian matrix is not square as there are 7 joint rotational speeds and 9 measured rotational velocities. The inverse relationship is used to compute the joint rotational speeds corresponding to the measured angular velocities. In Equation (3), $\dot{\vartheta }$ is an optimal (in terms of minimization of root mean square error) estimation of the joint rotational speeds and $J^{†}$ is the Moore–Penrose left pseudo-inverse of the Jacobian matrix J

(3) $$\dot{\vartheta }={\left(J^{T}J\right)}^{-1}J^{T}\Omega =J^{†}\Omega .$$

Joint angles ϑ can be estimated by integrating the joint rotational speed vector $\dot{\vartheta }$ as

(4) $$\vartheta =\vartheta_{0}+\int_{0}^{t}\dot{\vartheta }dt.$$

Due to the measurement noise, in particular gyroscope bias, in practical application, this approach can only be used for short periods of time and with known values of joint initial positions $\vartheta_{0}$. To compensate for the drift, the absolute orientation of the IMU can be estimated from the measured acceleration and magnetic field (e.g., q-method [27]). The orientation estimation error can then be computed as Δ and Equation (3) can be rewritten as

(5) $$\dot{\vartheta }=J^{†}\left(\Omega +K_{\Delta }\Delta \right),$$

where $K_{\Delta }$ is the error-correction gain matrix [26]. All quantities were computed relative to the IMU sensor placed on the trunk. From the known joint angles and joint rotational speeds hand position, hand orientation and their time derivatives can be computed.

The IMU attached on the trunk enabled estimating the trunk rotations around the three axes, as shown in Figure 1. The IMU data were processed with a Kalman filter [28] and the three trunk rotation angles (φ,ϑ,ψ) were computed from the IMU orientation.

In the following paragraphs, we describe methodology for movement analysis and computation of relevant parameters.

### 2.4. Movement Analysis

**Figure 2:**
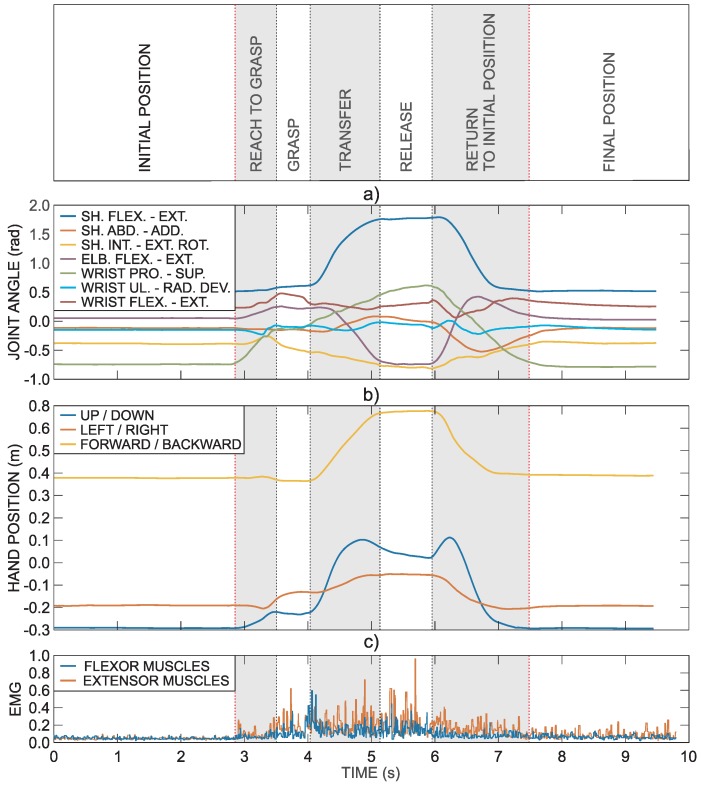
Hand and arm joint trajectories with the corresponding EMG activity. Hand and arm joint trajectories for the task that requires reaching for a block, transferring it onto the shelf and returning the hand to the initial position: (**a**) arm joint angles; (**b**) hand position relative to the trunk coordinate frame; and (**c**) normalized EMG activity of fingers and wrist flexor and extensor muscles.

Further explanation of used methodology is based on the task that requires grasping a block and transferring it onto a shelf (block with side length 100 mm). Figure 2 shows an example of healthy subject’s right arm trajectory and muscle activity during the task. The first three seconds represent the resting hand position on the table, which is followed by the movement sequence to grasp an object, transferring it onto the shelf in front of the subject and returning to the initial position. This specific task is divided into seven phases. The first and last phases require holding the initial and final positions. The five other phases are related to the execution of the task: reach to grasp phase, grasp phase, transfer phase, release phase, and return phase.

Figure 2a presents arm joint angles computed from the IMU data. The hand trajectory computed from the arm joint angles is presented in Figure 2b. The hand position is defined relative to the trunk reference coordinate frame $\left(x_{T},y_{T},z_{T}\right)$. Figure 2c shows normalized EMG activity of fingers and wrist flexor and extensor muscles as measured by the armband electrodes.

As explained in the above example, tasks are typically composed of movement periods and object manipulations. We refer to the movement periods as movement phases and object manipulations as manipulation phases; collectively, they are referred to as task phases.

The hand trajectory p(t) is the path p that the hand follows through space as a function of time *t*. The hand trajectory can be separated into path and speed analysis. In general, speed and form can be separated by parameterization with the arc length [29]. The arc length s(t) of time parameterized trajectory p(t) is given by

(6) $$s\left(t\right)=\int_{0}^{t}∥\dot{p}\left(t\right)∥dt.$$

**Figure 3:**
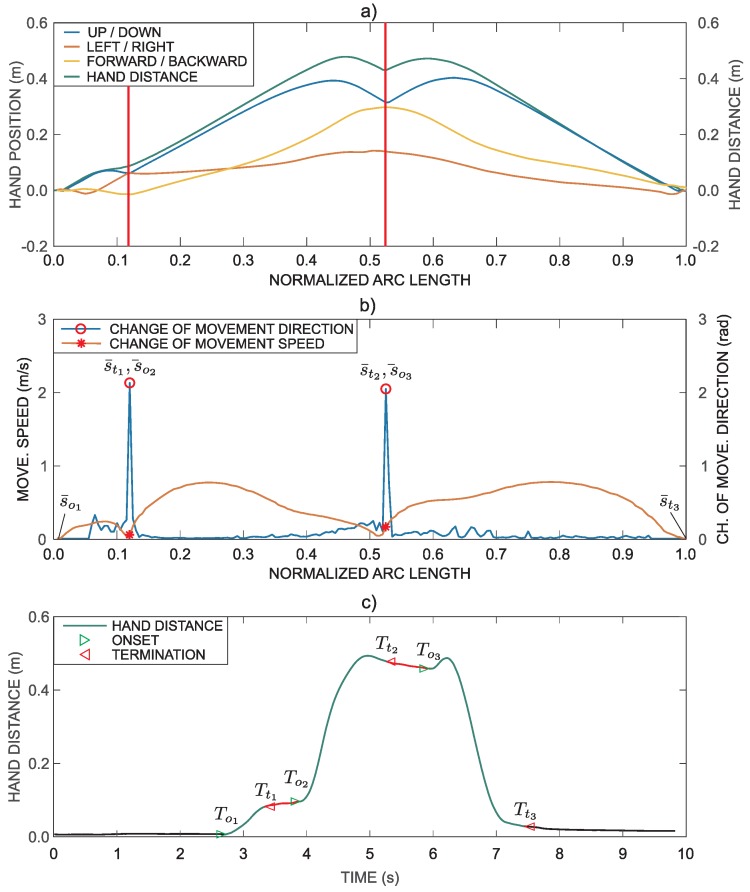
Movement segmentation for the task that requires grasping a block and transferring it onto a shelf. (**a**) Hand trajectory parameterized by the normalized arc length; (**b**) changes in movement direction and movement speed parameterized by the normalized arc length; and (**c**) transformation of segmented path into time domain. $T_{t_{i}}$ present the termination time and $T_{o_{i}}$ the onset time for phase *i*.

The length of the movement path *L* can be calculated as follows

(7) $$L=\int_{0}^{T_{m}}∥\dot{p}\left(t\right)∥dt,$$

where $T_{m}$ is the duration of the movement. Trajectory can be parameterized by the normalized arc length

(8) $$\bar{s}=\frac{s(t)}{L}$$

instead of time, resulting in $p(\bar{s})$, as shown in Figure 3a. It is also reasonable to resample the normalized arc length with a constant step $\Delta \bar{s}$. Simultaneously, the path in Figure 3a was transformed such that the movement starts from the zero position (all three coordinates are zero before the onset of movement) by subtracting the initial position. The distance from the initial position can be computed as

(9) $$d\left(\bar{s}\right)=∥p\left(\bar{s}\right)-p\left(0\right)∥.$$

By comparing the path in Figure 3a with the hand trajectory in Figure 2b, it can be observed that consecutive points in time domain, where movement velocity is zero, are transformed into a single point in the representation based on the normalized arc length.

With the aim to segment the task into phases, we focus on changes in movement direction and movement speed. The unit tangent vector $r(\bar{s})$ on the curve in point $p(\bar{s})$ can be computed as

(10) $$r\left(\bar{s}\right)=\frac{p^{\prime }\left(\bar{s}\right)}{∥p^{\prime }\left(\bar{s}\right)∥},\;$$

where $p^{\prime }\left(\bar{s}\right)$ is the derivative along the curve in point $p(\bar{s})$. If we consider two unit tangent vectors in two consecutive points on $p(\bar{s})$, $r(\bar{s})$ and $r(\bar{s}+\Delta \bar{s})$, the change of movement direction can be computed as the angle ν between tangent vectors in these two successive points

(11) $$\nu \left(\bar{s}\right)=\arctan\left(∥r\left(\bar{s}\right)\times r\left(\bar{s}+\Delta \bar{s}\right)\left|\right|,r\left(\bar{s}\right)\cdot r\left(\bar{s}+\Delta \bar{s}\right)\right),$$

where arctan in this case represents a four-quadrant inverse tangent function.

Figure 3b shows changes in movement direction $\nu (\bar{s})$. The same figure also shows movement speed $∥\dot{p}\left(\bar{s}\right)∥$ parameterized with the normalized arc length. The peak in the change of direction coincides with the minimum value of the movement speed.

As presented above, the task consists of three movement phases: reach to grasp phase, transfer phase, and return phase. The three movement intervals are clearly visible in Figure 3b. Thus, the task can be segmented into phases by considering hand position, changes in movement direction and movement speed. The relevant points are marked with markers in Figure 3b. A typical movement trajectory of a patient is often more complex in shape, which makes it more difficult for segmentation. All trajectories were automatically segmented based on the presented methodology. All segmentations were then visually inspected and corrected if necessary (in less than 15% of automatically segmented trajectories additional minor adjustments were performed to better correspond to visual observations). The computed points also represent approximate values for determining movement phases durations. The exact movement onset and termination times are derived from the movement trajectory represented in the time domain.

A point represented with the normalized arc length can correspond to several points in the time domain (all consecutive points where movement speed is close to zero). Thus, transformation from the arc-length parameterized domain to time domain is not unique. To match points on $p(\bar{s})$ with those on p(t), the minimum-distance algorithm was implemented based on [30] that searches for points on the time domain trajectory that are the closest to the arc-length parameterized trajectory points. To avoid finding similar positions at different times, search is limited to close proximity of the last found point in the time domain. The transformation is shown in Figure 3c.

Fine detection of movement onset and termination for each movement phase is based on a simple regression and follows the minimum acceleration with constraints model, in which the initial phase of the bell-shaped movement is modelled by a cubic power of the time [31]. Equation (12) summarizes the onset (termination) detection, where $x_{o}$ represents estimation of the initial static position before the movement onset, $\eta_{m}$ is the estimation of the initial jerk that best fits the data and $t_{o}$ is the estimation of the time instant of our interest

(12) $$x\left(t\right)=\left\{\begin{array}{cc} x_{o} & t\leq t_{o}\\ x_{o}+\frac{1}{6}\eta_{m}{\left(t-t_{o}\right)}^{3} & t_{o}\leq t\leq t_{1}=t_{o}+\Delta T. \end{array}\right.$$

Movement onset and termination were determined for each movement phase, as shown in Figure 3c. The onset of the task is determined at the time when a subject starts to move the hand, while the movement termination is determined when the hand is placed in the final position.

### 2.5. Quantification of Movement and Muscle Activity

Hand trajectory, joint angles, and muscle activity were measured for all tasks. Different parameters were determined for quality of movement analysis and comparison of arm motor functions within and between subjects.

#### 2.5.1. Movement Time

The time required to complete the task is the most obvious parameter to observe. Within the ARAT, a subject starts the task when ready and the examiner estimates instances of movement onset and termination. With standard measurement task duration is estimated using a stopwatch, evaluation of task phases duration is not possible. With the instrumented measurement and movement segmentation, phase durations can be computed for each phase separately as

(13) $$T_{m_{i}}=T_{t_{i}}-T_{o_{i}},$$

where $T_{m_{i}}$ is the movement time for phase *i* computed as the difference between the termination time $T_{t_{i}}$ and the onset time $T_{o_{i}}$ for phase *i*. Movement time for the complete task is represented with $T_{m}$ defined as the difference between the termination time of the last task phase and the onset time of the first task phase.

#### 2.5.2. Movement Smoothness

Smooth trajectories are characteristic of coordinated human movements and stroke patients’ movements become smoother with recovery [32]. Movement smoothness can be estimated with different parameters [33,34], including various jerk-based approaches.

Typically, jerk is determined as the third derivative of position over time. Different methods for computing jerk were evaluated for the scope of this study: (1) the third derivative of hand position over time; (2) the first derivative of hand acceleration as measured by the accelerometer inside the IMU placed on the back of the hand over time; and (3) the second derivative of the angular velocity as measured by the gyroscope inside the IMU placed on the back side of the hand. Jerk computed from the gyroscope data are referred to as rotational jerk. The rationale for such jerk definition is that rotational jerk takes into account not only smoothness of hand position trajectory, but also stability of hand orientation. Change of hand position is a result of rotational movements in individual arm joints. Thus, smoothness of hand position trajectory is also reflected in the rotational jerk. The comparative analysis showed that the rotational jerk results enable differentiation between different conditions (the analysis also included non-jerk based parameter spectral arc length (SAL) [35]).

Rotational jerk was first calculated for all three rotational axes and then the hand rotational jerk magnitude was calculated as the norm of the rotational jerk vector. The jerk index parameter [36], calculated as a logarithm of the jerk, averaged over the movement duration and normalized with respect to the amplitude and duration of the reaching movement [37,38] was used to calculate the rotational jerk index

(14) $$\eta_{rot_{i}}=\log\sqrt{\frac{{\left(T_{t_{i}}-T_{o_{i}}\right)}^{5}}{2\theta^{p}}\int_{T_{o_{i}}}^{T_{t_{i}}}{∥\frac{d^{2}\omega \left(t\right)}{dt^{2}}∥}^{2}dt},$$

where $\eta_{rot_{i}}$ represents the rotational jerk index for phase *i*, ω(t) is the hand angular velocity vector and parameter θ normalizes jerk index with the angular displacement of the rotational movement to produce a dimensionless result. In most ARAT tasks (except task of pouring water from one glass to another), the subject is required to keep the orientation of the hand. Thus, normalization was performed with the angular displacement $\theta^{0}=1$. The rotational jerk index was computed for the task and for each movement phase separately.

#### 2.5.3. Similarity of Hand Trajectories

Movement trajectories of the affected arm can be compared to those of healthy subjects or to trajectories of the unaffected arm. Different trajectory similarity measures can be implemented for the given task [39]. Several approaches were compared for the purpose of this study (dynamic time warping based approaches, edit distance based approaches, Euclidian distance, Hausdorff distance, and Frechet distance). Finally, a weighted combination of two parameters was selected.

**Figure 4:**
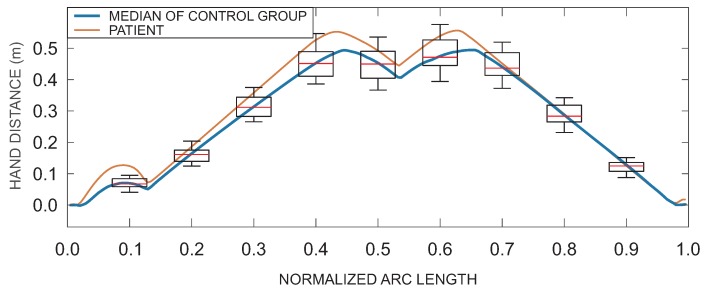
Hand distances for healthy subjects and a single patient for the task that requires grasping a block and transferring it onto a shelf. Median hand distance with box plots for control group (blue) and an example of patient’s hand distance (red).

The first parameter is the Euclidian distance, a standard trajectory similarity measure. As trajectories are parameterized with the normalized arc length, Euclidian distance can be computed for each point along the normalized arc length. The parameter is defined as

(15) $$D_{i}=\sqrt{\frac{1}{{\bar{s}}_{t_{i}}-{\bar{s}}_{o_{i}}}\int_{{\bar{s}}_{o_{i}}}^{{\bar{s}}_{t_{i}}}{∥p\left(\bar{s}\right)-p_{r}\left(\bar{s}\right)∥}^{2}d\bar{s}},$$

where $D_{i}$ is the root mean square Euclidian distance for the phase *i* and $p_{r}\left(\bar{s}\right)$ is the reference trajectory defined from the group of healthy subjects (control group) as the median trajectory for a specific task parameterized by the arc length, ${\bar{s}}_{o_{i}}$ and ${\bar{s}}_{t_{i}}$ are the movement onset and termination of phase *i* expressed with the normalized arc length (see Figure 3b). As an example, Figure 4 shows the median (reference) hand distance (blue) with box plots representing distribution of hand distances for healthy subjects and an example of patient’s hand distance (red) (similar curves were obtained for hand position, but are not shown for simplicity of presentation).

The second trajectory similarity parameter is related to changes of movement direction $\nu (\bar{s})$ during task execution (see Equation (11)). It can be computed as a root mean square value of $\nu (\bar{s})$ during the movement period *i*

(16) $${\hat{\nu }}_{i}=\frac{1}{\pi }\sqrt{\frac{1}{{\bar{s}}_{t_{i}}-{\bar{s}}_{o_{i}}}\int_{{\bar{s}}_{o_{i}}}^{{\bar{s}}_{t_{i}}}\nu^{2}\left(\bar{s}\right)d\bar{s}}$$

and normalized with π, which represents the largest possible change in direction.

Trajectory similarity parameter is defined as

(17) $$\chi_{i}=\alpha D_{i}+\beta {\hat{\nu }}_{i},$$

where $\alpha =0.5\;m^{-1}$ and β=0.5 are the weights. Equal weights were selected as $D_{i}$ and ${\hat{\nu }}_{i}$ have similar magnitudes in measurements.

#### 2.5.4. Trunk Stability

When a therapist evaluates a patient with ARAT, movement of the trunk in forward direction is considered a criterion between Score 2 and Score 3. To determine trunk stability during the task execution, we considered all three rotation angles (see Figure 1) by computing the trunk stability index $\rho_{i}$ as a maximum of the root mean square value of all angular deviations from the initial position (φ(0),ϑ(0),ψ(0))

(18) $$\rho_{i}=\max_{\bar{s}\in \left[{\bar{s}}_{o_{i}},{\bar{s}}_{t_{i}}\right]}\left(\sqrt{{\left(\varphi \left(\bar{s}\right)-\varphi \left(0\right)\right)}^{2}+{\left(\vartheta \left(\bar{s}\right)-\vartheta \left(0\right)\right)}^{2}+{\left(\psi \left(\bar{s}\right)-\psi \left(0\right)\right)}^{2}}\right).$$

Compensatory movements parameter $\rho_{i}$ is mostly affected by the trunk displacement in the sagittal plane (angle $\vartheta_{i}$). Parameter $\rho_{i}$ was found to better differentiate between different conditions than angle $\vartheta_{i}$ only.

#### 2.5.5. Muscle Activity

Eight EMG electrodes were equidistantly positioned around the subject’s forearm in the proximity of the elbow joint. Electrodes were split for measurement of fingers and wrist flexor and extensor muscle activity [40]. The analysis showed coactivation of flexor and extensor muscles during grasping. Thus, EMG analysis was performed by taking into account all eight electrodes by computing the RMS value as

(19) $${\hat{w}}_{i}=\sqrt{\frac{1}{T_{m_{i}}}\int_{T_{o_{i}}}^{T_{t_{i}}}\left(\sum_{n=1}^{8}w_{n}^{2}\left(t\right)\right)dt},$$

where $w_{n}\left(t\right)$ is the output of the electrode number *n*.

The signal was then normalized relative to the maximum value ${\hat{w}}_{MAX}$ computed from Equation (19), with EMG data from the dynamometry. The normalized EMG value ${\hat{w}}_{i}$ for movement period *i* was computed as

(20) $${\hat{w}}_{n_{i}}=\frac{{\hat{w}}_{i}}{{\hat{w}}_{MAX}}.$$

#### 2.5.6. Ergonomic Issues and Effect of Sensor Limitations on Parameter Values

All sensors are lightweight, battery powered, and wireless. Therefore, they do not obstruct a patient’s movement. Sensor placement takes about a minute for a skilled therapist. Accuracy of the angle measurement based on the IMU sensors is less than 2.5° in static conditions and less than 5° in dynamic conditions. Patients’ movements are relatively slow; therefore, quasi-static conditions can be assumed. Estimation of arm kinematics can be affected by placement of IMU sensors. However, by following the described protocol for sensor placements and by taking into account the redundancy of measurements [26], IMU-based measurement system does not affect movement analysis quality. Movement time, movement smoothness, trunk stability, and muscle activity do not depend on arm trajectory. Similarity of hand trajectories depends on computed trajectories. However, the parameter estimates differences in movement shape not position, thus additionally reducing uncertainty related to IMU sensor attachment. Muscle activity analysis is affected by the placement of EMG electrodes and the quality of acquired signals. Signals from all eight bracelet sensors were combined when estimating muscle activity. Thus, bracelet orientation does not affect measurement outcome. Signals were normalized to values from dynamometry, which enables comparison between multiple subjects. However, relatively low sampling rate (200 Hz) and low quantization resolution (256 values) should still be considered when assessing muscle activity.

### 2.6. Statistical Analysis

The Mann–Whitney test was used to analyse differences in kinematics and EMG parameters between different groups of subjects. The test was computed for all parameters, for each movement phase, as well as for the whole task. Data were allocated into four groups. The first group represents the healthy subjects (H) in which no significant differences were observed between performance with dominant and non-dominant arms. Thus, data from the left and right arm were combined for further analysis. Patients who suffered stroke were grouped based on their clinical scores obtained for each task. Only patients who were able to complete the task were included in the analysis (patients that obtained ARAT Score 2 or 3). The second group represents data from patients’ unaffected arm (UAF), if patients obtained maximal score (ARAT Score 3) for their performance. In 10% of all tasks, patients did not obtain maximal score for their unaffected (less affected) arm and those data were not used in the analysis. The third group includes patients who performed the task with their affected arm and obtained Score 3 (AF-3). We refer to these results as *normal task execution*. The fourth group includes patients who completed the task with their affected arm but obtained Score 2 (AF-2). These results aree referred to as *moderate task execution*. Patients who obtained Score 0 or 1 for ARAT due to being unable to start or complete the task were excluded from further analysis (*uncompleted task execution*). Their movement trajectories differ significantly from the trajectories of the completed task and cannot be evaluated with the set of defined parameters. Incomplete movement analysis is beyond the scope of this study.

Relations between the ARAT clinical scores and values from the measurement system were analysed using the Spearman’s rank correlation coefficients ($\rho_{S}$) for all ARAT subtests, parameters and movement phases. In this analysis, the patients were not grouped according to their performance. The analysis was performed for the patients’ affected arm.

Analysis was performed with significance levels set to p≤0.05.

## 3. Results

**Figure 5:**
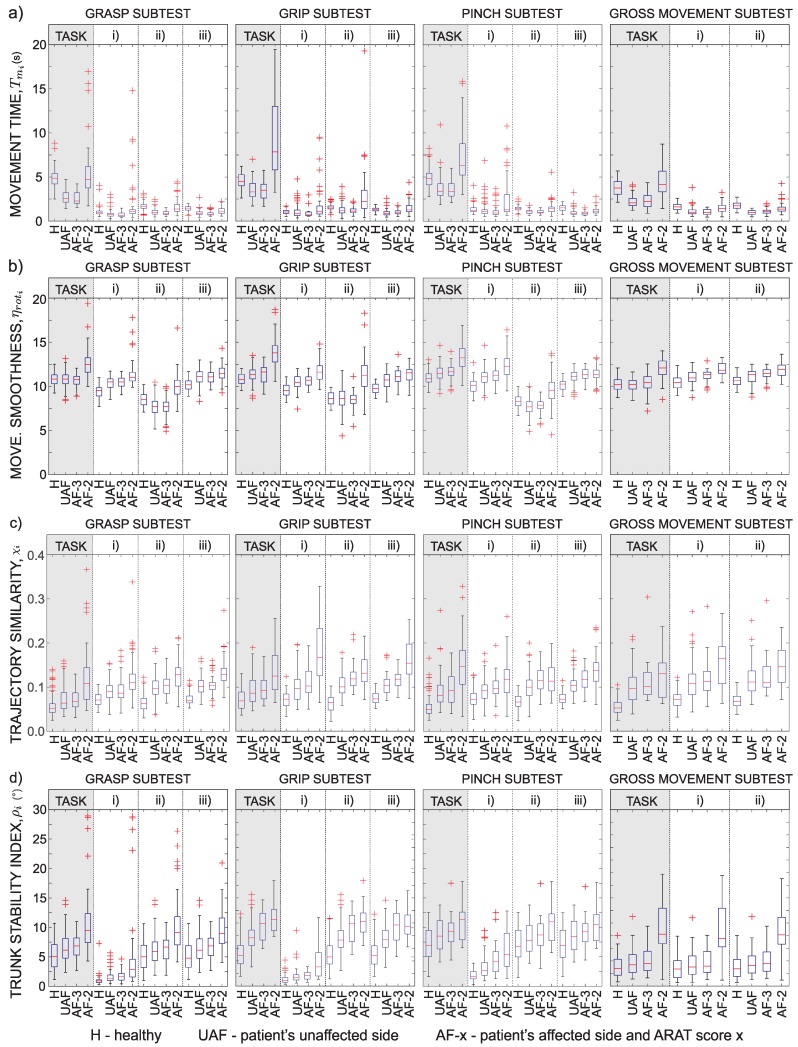
Quantification of movement during ARAT tasks execution. Parameters for movement analysis: (**a**) movement time $T_{m_{i}}$; (**b**) movement smoothness represented as rotational jerk index $\eta_{rot_{i}}$; (**c**) trajectory similarity $\chi_{i}$; and (**d**) trunk stability index $\rho_{i}$. Quantities are presented for the task (grey area): (i) reach to grasp phase; (ii) transfer phase; and (iii) return phase. Presented tasks from left to right are: (1) grasp subtest (different blocks, cricket ball, sharpening stone); (2) grip subtest (different alloy tubes and washer); (3) pinch subtest (marbles, bearing balls); and (4) gross movement subtest. Presented groups are: H, healthy; UAF, patient’s unaffected side; and AF-*x*, patient’s affected side and Score *x*.

Not all patients were able to complete all tasks. If the patient was not able to complete the task, data for the specific patient and the specific task were excluded from analysis. In a limited number of tasks, quality of acquired signal was too low (due to loss of data packets on a wireless network) and such tasks were excluded from analysis as well. In total, 1440 task executions were measured and 1150 were included in the analysis (290 measurements were excluded due to the above-mentioned reasons). Detailed kinematics results are presented as box plots for four ARAT subtests in Figure 5. Figure is organized as a 4×4 matrix with rows representing different parameters (movement time, rotational jerk index, trajectory similarity, and trunk stability) and columns representing different subtests (from left to right): the first set of box plots represents results for the grasp subtest that requires putting different blocks, cricket ball, and sharpening stone on the shelf; the second set of box plots represents grip subtest that requires displacement of two different sized alloy tubes and washer from the starting peg on a plank to a target peg on a plank; the third set of box plots represents pinch subtest that requires putting marbles and bearing balls on the shelf; and the last set of box plots represents gross movement subtest that requires the subject to move the hand towards different parts of head.

Figure 5a shows movement time $T_{m_{i}}$ for ARAT subtests and movement phases. Healthy subjects were asked to perform the movement with normal speed in order to focus more on the trajectory of movement. Thus, their movement times are often longer than those of patients that were asked to execute the task as quickly as possible. Results indicate that the performance of patients with normal task execution is similar to the performance with their unaffected arm. On the other hand, movement time increases for patients with moderate task execution. Patients that were not able to complete the task within 60 s were excluded from this study.

Figure 5b shows rotational jerk index $\eta_{rot_{i}}$. Index is typically the lowest for healthy subjects. Normal execution of patients with their affected arm results in similar index values as with their unaffected arms. The index increases for patients with moderate execution. The rotational jerk index was found similar to the position-based jerk index. However, though not presented here, rotational jerk better differentiates between the groups for the performed ARAT tasks.

Figure 5c shows the hand trajectory similarity measure $\chi_{i}$. The reference trajectory for computing the Euclidian distance $D_{i}$ was computed from trajectories of healthy subjects. Thus, $\chi_{i}$ values for group *H* are the lowest. Patients with moderate task execution show significant increase of similarity measure $\chi_{i}$. This is also the case for both trajectory similarity parameters, $D_{i}$ and ${\hat{\nu }}_{i}$, although not presented here. Differences in the similarity measure $\chi_{i}$ can also be observed in performance of patients with their unaffected arm and normal task execution with the affected arm.

The trunk stability index $\rho_{i}$ is presented in Figure 5d. The index is sensitive to trunk movements of less than one degree. Healthy subjects had the highest trunk stability. However, for complex tasks, trunk movements of up to $10^{\circ }$ were measured with healthy subjects, although they were asked to execute the task with normal velocity while maintaining their trunk posture. Patients with moderate task execution show significantly lower trunk stability.

**Figure 6:**
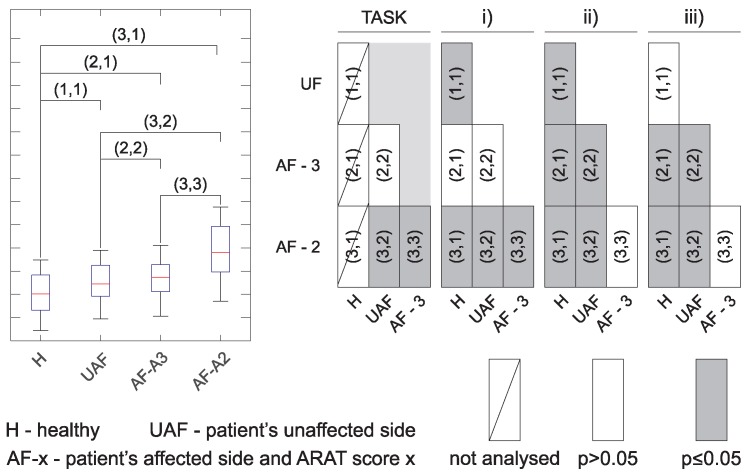
Explanation of presentation of statistical significance. Left side shows a box plot for one of the movement phases; in the parenthesis are indicated possible comparisons between different groups. Significance levels are presented in a graphical form on the right side. The first column in each phase compares healthy subjects to the other groups, while the last row compares patients with moderate task execution to the other groups.

Similar analysis as presented in Figure 5 was performed for all tasks together with the analysis of statistically significant differences. Results are presented in a compressed form as explained in Figure 6. The left side in Figure 6 shows a box plot as used to present results for different movement phases in Figure 5. In the parenthesis are indicated possible comparisons between different groups. The significance levels are then presented in a graphical form as shown on the right side of Figure 6. Empty boxes indicate non-significant differences (p>0.05), grey boxes are for p≤0.05, and boxes with diagonal line indicate cases where statistical significance was not computed (e.g., movement times for healthy subjects were not considered in the analysis).

**Figure 7:**
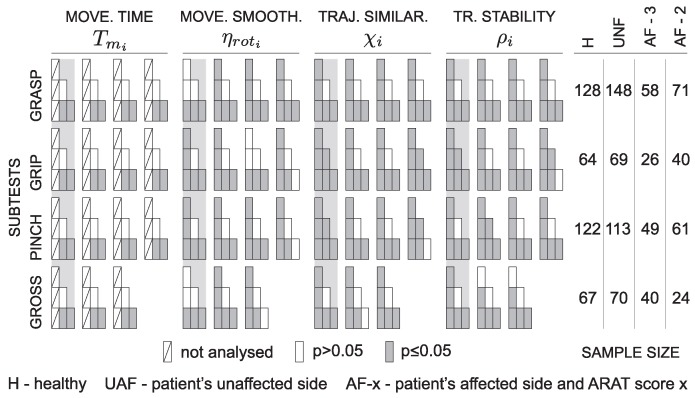
Statistical significance for the subtests. Four subtests, grasp, grip, pinch and gross, quantified with four parameters: movement time, movement smoothness, hand trajectory similarity, and trunk stability. Presentation is organized as explained in Figure 6. The columns on the right side represent the number of trajectories included in statistical analysis for each condition.

Due to the high number of parameters, the significance levels are presented separately in Figure 7 along with the actual sample size for each ARAT subtest. Quick overview shows significant differences between healthy subjects and the other groups for all tasks and all parameters (except movement time, as already explained above). Significant differences are also visible between patients with moderate execution and all other groups for most of the parameters.

**Figure 8:**
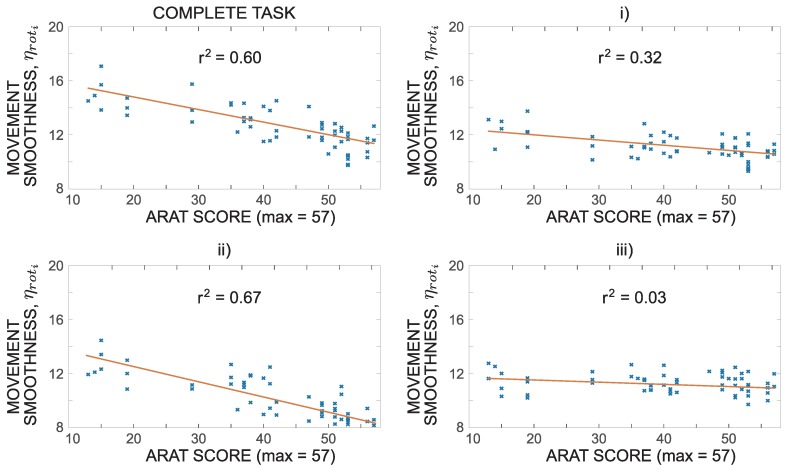
Relation between ARAT score and movement smoothness represented as patient’s affected arm rotational jerk index $\eta_{rot_{i}}$ computed for the grip subtest and its three phases.

In Figure 7, patients were grouped based on their clinical scores obtained for each task. Numerical quantification of movement was additionally compared to the total ARAT scores obtained by each patient. Figure 8 shows an example of such analysis for movement smoothness represented as patient’s affected arm rotational jerk index $\eta_{rot_{i}}$ computed for the grip subtest and its three phases. A monotonic relationship between the movement smoothness and ARAT scores can be observed. For this particular case, the four plots are augmented with linear regression lines with the corresponding $r^{2}$ coefficient of determination.

Table 1 summarizes results of the Spearman’s rank correlation coefficients. All, except correlations indicated with minor italic font, have p≤0.05. Negative signs of the coefficients indicate higher parameter values associated with lower ARAT scores. Mostly strong correlations ($\rho_{S}\geq 0.60$) were observed for parameters movement time $T_{m_{i}}$ and movement smoothness $\eta_{rot_{i}}$ for the complete task as well as the first and second movement phase. Moderate correlations ($\rho_{S}\geq 0.40$) were identified for trunk stability index $\rho_{i}$ for grasp and gross subtests and trajectory similarity $\chi_{i}$ for grasp and grip subtests. Weak ($\rho_{S}<0.40$) or non-significant correlations were observed for trunk stability index for grip and pinch subtests and trajectory similarity for pinch and gross movement subtests.

**Figure 9:**
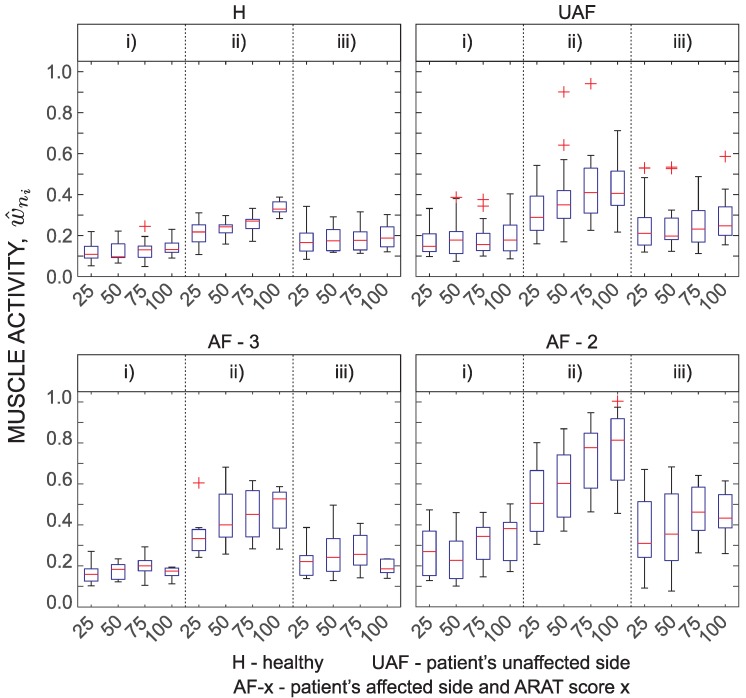
Muscle activity ${\hat{w}}_{n_{i}}$ for four grasp tasks. Four blocks: Side length 25 mm, 50 mm, 75 mm, and 100 mm. Three phases: (i) reach to grasp phase; (ii) transfer phase; and (iii) return phase. Presented groups are: H, healthy (top-left); UAF, patient’s unaffected side (top-right); AF-3, patient’s affected side with Score 3 (bottom-left); and AF-2, patient’s affected side with Score 2 (bottom-right).

Results of fingers and wrist muscle activity analysis are presented in Figure 9 as a normalized EMG parameter ${\hat{w}}_{n_{i}}$. Presentation of EMG activity differs from the above presentations. Tasks were not grouped. Here, four grasp tasks that require transfer of blocks of various side lengths (25 mm, 50 mm, 75 mm, and 100 mm) were selected for presentation. EMG is mostly related to the grasping activity. Therefore, the parameter ${\hat{w}}_{n_{i}}$ changes between different task phases. Consecutively, the results are only presented for each movement phase and not for the entire task. Expected differences can be noticed between different phases. The most interesting part is the second movement phase that represents transfer of the held block onto the shelf. The normalized EMG activity monotonically increases with the size of the block. Interestingly, ${\hat{w}}_{n_{i}}$ differs also between subject groups with the lowest normalized EMG activity detected for healthy subjects and the highest normalized EMG activity measured for patients with moderate task execution.

**Figure 10:**
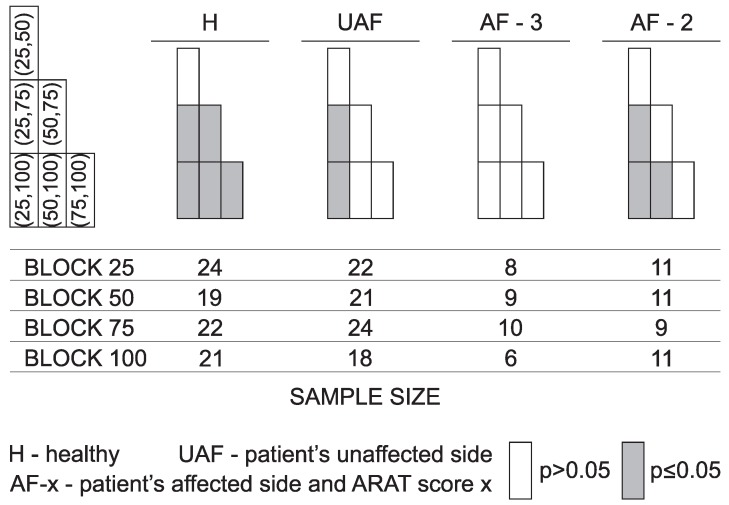
Statistical significance for muscle activity ${\hat{w}}_{n_{i}}$ for four grasp tasks. Four blocks: Side length 25 mm, 50 mm, 75 mm, and 100 mm during the transfer phase. Presented groups are: H, healthy (first matrix); UAF, patient’s unaffected side (second matrix); AF-A3, patient’s affected side with Score 3 (third matrix); and AF-A2, patient’s affected side with Score 2 (fourth matrix).

Figure 10 shows significance levels for the transfer phase (second phase) corresponding to the muscle activity presented in Figure 9.

## 4. Discussion

The presented wearable measurement system is non-invasive and does not disturb subject’s movement. The benefit of using sensor based technologies is the possibility of quantification of arm function with different parameters. At the same time, instrumented measurements provide a more detailed insight into the limitations of upper limb use through analysis of individual task phases [15]. Inter-subject differences were analysed by comparing groups of healthy subjects, patients with normal task execution, and patients with moderate task execution. Inter-arm analysis was based on comparison between the unaffected and the affected limb execution in patients after stroke. Analysis of statistically significant differences between groups presented in Figure 7 indicates relevant differences between healthy subjects and all three groups of patients. Differences were greater for patients with moderate execution (ARAT Score 2) compared to other groups. In most of the tasks and for most of the computed parameters, there were no differences between the task execution with patients’ unaffected arm and the patient’s arm scored with ARAT Score 3.

Tasks were combined into groups of ARAT subtests for statistical analysis. Although tasks are similar in terms of movement trajectories, they still differ in the required level of motor performance. On the other hand, grouping resulted in less information to present and more importantly, more trajectories were available for analysis, thus increasing the statistical power.

Movement time is a well-established arm assessment parameter. In standard clinical assessment, movement time is measured for the complete task execution. Studies show that ARAT score strongly correlates with movement time [17]. With the segmentation of task into phases, duration can be computed for each phase. Tasks that require grasping and transferring of an object can be separated into phases that are related to arm movement and object manipulation with fingers (fine motor abilities). Analysis presented in this study can expose different limitations in arm motor functions. However, to establish pre-grasp and manipulation phase kinematics, detection of fingers movement is missing. Based on the analysis of movement times for the complete task and movement phases (Figure 7), it is not possible to detect differences between patient’s task execution with the unaffected arm and the normal task execution with the affected arm (ARAT Score 3). However, significant differences were observed for patients with moderate task execution compared to the other groups. Differences were significant for all subtests. Differences in movement times were observed for the complete task as well as for each movement phase. Healthy subjects were not included in the analysis of movement times since they did not execute the task with their maximal velocity.

The above-mentioned differences between groups are clearly seen in Figure 5a for all task groups. Reaching for a marble (pinch subtests) requires finer motor abilities than reaching for a block (grasp subtest). Therefore, the first phase is slightly longer than in the grasp subtest. On the contrary, putting a block onto the shelf is more demanding and the second phase is slightly longer for the grasp subtest. There are no differences in movement time during the return phase for the two subtests.

Movement smoothness was analysed with various parameters. As shown in Figure 5b and Figure 7, the selected parameter, rotational jerk index, differentiates well between healthy subjects and the other groups. The parameter differentiates also between patient’s moderate task execution and the other conditions. The rotational jerk index does not indicate differences between movements performed with the patient’s unaffected arm and normal task execution with the affected arm (ARAT Score 3). Similar to the movement time, differences in movement smoothness were found for the complete task as well as for each movement phase. Both movement time and movement smoothness were found as valuable parameters that are able to discriminate motor performances of persons with upper limb impairment and healthy subjects [18].

Parameter $\chi_{i}$ was introduced as a hand trajectory similarity measure. The parameter combines two relevant characteristics of a trajectory: (1) deviations from the *ideal* path that healthy subjects follow; and (2) changes in movement direction. As the parameter indicates similarities between trajectories, low $\chi_{i}$ values (indicating similar trajectories) would be expected for gross movement tasks and high values for more complex tasks that require better motor coordination for achieving optimal movement trajectories. Results presented in Figure 5c and Figure 7 demonstrate excellent differentiation between subject groups based on the trajectory similarity measure. The parameter differentiates quality of movement in all phases. Mostly no significant differences were observed between the unaffected arm and the affected arm execution with ARAT Score 3.

Movement quantification with IMUs on arms and sternum compared to using one IMU on the wrist [12] enables evaluation of arm movements relative to the sternum and evaluation of trunk movements. Arm movement is often accompanied with trunk displacement to facilitate larger range of motion or to compensate for functional limitations of upper limbs. The setup of ARAT measurements does not require trunk movement to increase the range of motion for completing the task. Thus, measured displacements can be mainly attributed to compensatory movements. Trunk stability index presented in Figure 5d and Figure 7 shows significant differences between different groups as well as between different movement phases. The transfer and return phases indicate similar displacements in absolute values though typically in opposite directions. It should be noted that trunk displacements that occasionally exceed $10^{\circ }$ were detected in healthy subjects as well. The group of patients with moderate task execution (ARAT Score 2 ) significantly differs from the other groups for almost all tasks.

Spearman’s rank correlation coefficients $\rho_{S}$ were computed to analyse relations between numerical quantifications and ARAT scores (summarized in Table 1). Coefficients were determined for all ARAT subtests (tasks and movement phases). Out of 60 computed Spearman’s coefficients 8 were not statistically significant. Movement time $T_{m_{i}}$ and movement smoothness $\eta_{rot_{i}}$$\rho_{S}$ for complete task execution show strong correlation with patients’ ARAT scores indicating that these two parameters have higher impact on clinical assessment compared to trajectory similarity $\chi_{i}$ and trunk stability index $\rho_{i}$. Trajectory similarity shows moderate correlations with ARAT scores for two subtests (grasp and grip), weak correlation for pinch subtest and non-significant values for gross movement subtest. Weak to moderate correlations were expected as trajectory similarity cannot be quantitatively assessed by the clinician through direct visual analysis of a movement. Moderate correlation coefficients were observed for trunk stability index $\rho_{i}$ for grasp and gross subtests, while low correlation coefficients were observed for grip and pinch subtests. As trunk stability is considered in ARAT assessment, higher correlations would be expected. However, trunk movement is relatively challenging to quantify from visual observations. In previous studies [14], smooth movements were significantly associated with the clinical scores, and the sternum accelerometer showed that linear regression models were able to detect compensatory trunk movement associated with a lower clinical scores. Interestingly, in our study, trunk stability and trajectory similarity were found to differentiate between groups of subjects even better than movement smoothness (Figure 5 and Figure 7), however they do not strongly correlate with ARAT scores. It should be noted that the clinical score combines measured movement time and subjectively observed movement characteristics. Therefore, very strong correlations between measured parameters and ARAT scores are unlikely.

Analysis of correlations coefficients $\rho_{S}$ through different movement phases reveals some relevant observations. For movement time and movement smoothness, the highest correlations were observed for the most challenging movement phase (typically transfer phase). Movement time was also found to moderately or strongly correlate in other phases. On the other hand, movement smoothness is relevant in the first phase (reach to grasp), but not in the last phase (return to initial position), which was the least challenging for the subjects. For trunk stability, the highest correlation coefficients $\rho_{S}$ were observed for the first movement phase indicating that the relevant trunk movement occurs already early during the execution of the task. Trajectory similarity for grasp and pinch subtests is most relevant in the first phase (reach to grasp) and in the second, most challenging phase for grip subtest. In the second and third phases of ARAT grip subtest, the trunk movements correlation coefficient $\rho_{S}$, as well as in complete task, first and second phases of ARAT gross movement subtest, the trajectory similarity correlation coefficient $\rho_{S}$ were not statistically significant indicating high variability of results.

The overall observation is that the four proposed parameters for movement assessment (movement time $T_{m_{i}}$, movement smoothness represented as rotational jerk index $\eta_{rot_{i}}$, trajectory similarity $\chi_{i}$, and trunk stability index $\rho_{i}$) differentiate between patients with different motor functions. The two parameters that are considered during clinical assessment (movement time and movement smoothness) strongly correlate with the patients’ ARAT scores. Trajectory similarity correlates weak to moderate with ARAT scores. However, this does not reduce its value as a tool for assessment of patients’ motor performance, which is the case also for trunk stability.

When performing activities of daily living, grasping and manipulation of objects is important. Although the main focus of this study was movement trajectories, ARAT also assesses grasping and manipulation functions. These functions were not measured directly. Previous studies show F/M ratio of EMG signal can be used to indicate spasticity [11,41]. In our study EMG of fingers and wrist flexor and extensor muscles was used as an indication of grasping (Figure 9). To compare between different conditions, we focused on four ARAT tasks that require grasping of blocks of various sizes and weights. Specifically, the focus was on the transfer phase when the subject moves the block from the table onto the shelf. Results demonstrate differences between grasp and no-grasp phase for blocks of all sizes. Measured muscle activity is also an indication of the power required for grasping. The normalized muscle activation indicates that patients with more affected arm (ARAT Score 2) activate muscles almost to their limit when grasping the largest block. On the other hand, for healthy subjects, this ratio was approximately one third. Differences between muscle activity when grasping blocks of different sizes were significant for healthy subjects. Differences between the patient groups were less significant. This can be partially attributed to small statistical power of the sample size. In summary, measurement of EMG activity during ARAT task provides complementary information to the analysis of trajectories and can be used as an indication of grasping activities.

Unlike previous studies [12,19,21], the present study quantifies patients’ movement during all ARAT tasks based on IMU and EMG measurements with the same set of five parameters for the complete task and for each movement phase. The five parameters enable differentiation between patients allocated to different groups according to their clinical score. In the current study, EMG and kinematic data were observed independently for specific movement phases. However, the same measurement setup can be used for analysis of activities of daily living where coordination of limb movement and grasping is relevant. Analysis of movement kinematics and finger and wrist EMG activity can provide insight into the actual use of the affected and unaffected upper limbs.

## 5. Conclusions

Instrumented ARAT assessment enables quantification of movement parameters and might provide a better insight into the arm motor function. In this study, patient’s movement was quantified with four parameters: (I) movement time; (II) movement smoothness; (III) hand trajectory similarity; and (IV) trunk stability. An additional parameter was introduced as an indicator of grasping activity.

The presented movement parameters mostly differentiate between different subject groups for all movement phases as well as for the complete tasks. Patients’ movements with the unaffected and the affected arm with ARAT Score 3 are similar to the level that cannot be distinguished from the kinematic analysis for most of the test conditions. Differences in numerical parameters are significant between patient groups that obtained different clinical scores. Muscle activity is a good indicator of grasping activity and the level of grasping force.

Analysis of relations between overall ARAT scores and numerically quantified movement parameters indicates strong correlations between patient’s ARAT scores and movement time as well as movement smoothness. Weak to moderate correlations were observed for parameters that describe hand trajectory similarity and trunk stability. Movement time and movement smoothness are strongly associated with clinical scores, while similarity of hand trajectories cannot be quantitatively determined from visual observations. On the other hand, weak to moderate correlations for trunk stability were not entirely expected as trunk movement is considered during the clinical assessment and can also be quantified from visual observations.

Segmentation of movement into phases enables a more detailed quantification of specific arm function limitations. This study was focused on the movement phases. However, for tasks that require grasping and manipulation of objects, additional analysis could also be performed for these phases. Such analysis would provide an insight into gross and fine arm and hand motor abilities.

The presented approach clearly also has potential limitations. These are related to the technological limitations of sensors as well as methodological approaches. Limitations related to sensors are their number, positioning, and disturbances. Fewer sensors are typically better for everyday use. On the other hand, a full set of seven sensors provides detailed information about upper limb movement. Positioning of sensors affects computed limb trajectories. This affect can be reduced by careful placement of sensors and a robust algorithm that exploits redundancy of measurements. Ferromagnetic materials might disturb magnetometer output and environment cluttered with wireless networks might affect wireless transmissions of data. From the methodological perspective, the most critical is movement segmentation into specific movement phases. Due to complex and jerky patient movements, sometimes even manual segmentation becomes challenging. Automatic segmentation is mostly affected by high amplitude jerky movement near the final position, which makes it difficult to detect the actual movement termination. Movement time is the most affected parameter resulting from erroneous segmentation. Other parameters are typically more robust to movement segmentation as they do not depend on the exact onset and termination times.

## Figures and Tables

**Table 1:** Spearman’s rank correlation coefficient $\rho_{S}$ for four ARAT subtest (grasp, grip, pinch, and gross) and four analysed parameters (movement time $T_{m_{i}}$, movement smoothness represented as rotational jerk index $\eta_{rot_{i}}$, trajectory similarity $\chi_{i}$, and trunk stability index $\rho_{i}$). $\rho_{S}$ for the task is shown in grey area, followed by (i)–(iii) for each movement phase. Values written with minor italic font are not statistically significant (p>0.05).

	GRASP SUBTEST				GRIP SUBTEST				PINCH SUBTEST				GROSS MOVEMENT SUBTEST		
	TASK	(i)	(ii)	(iii)	TASK	(i)	(ii)	(iii)	TASK	(i)	(ii)	(iii)	TASK	(i)	(ii)
$T_{m_{i}}$	−0.77	−0.61	−0.60	−0.50	−0.81	−0.54	−0.68	−0.61	−0.62	−0.43	−0.66	−0.38	−0.64	−0.64	−0.45
$\eta_{rot_{i}}$	−0.80	−0.41	−0.78	−0.22	−0.76	−0.43	−0.82	*−0.23*	−0.67	−0.47	−0.69	−0.30	−0.64	−0.48	*−0.22*
$\chi_{i}$	−0.47	−0.49	0.36	−0.28	−0.62	−0.38	−0.66	−0.39	−0.41	−0.29	−0.26	−0.33	*0.03*	*0.02*	*−0.03*
$\rho_{i}$	−0.46	−0.46	−0.43	−0.37	*−0.15*	−0.33	*−0.09*	*−0.06*	−0.29	−0.39	−0.27	−0.26	−0.57	−0.55	−0.59

## Abbreviations

The following abbreviations are used in this manuscript:

ARAT	Action research arm test
AF	patient’s affected side
EMG	electromyography
H	healthy subjects
IMU	inertial measurement unit
SAL	spectral arc length
UAF	patient’s unaffected side
WMFT	Wolf motor function test
